# Organic Cation Transporter 1 (OCT1) mRNA expression in hepatocellular carcinoma as a biomarker for sorafenib treatment

**DOI:** 10.1186/s12885-016-2150-3

**Published:** 2016-02-12

**Authors:** Daniel Grimm, Jonas Lieb, Veronika Weyer, Johanna Vollmar, Felix Darstein, Anja Lautem, Maria Hoppe-Lotichius, Sandra Koch, Arno Schad, Jörn M. Schattenberg, Marcus A. Wörns, Arndt Weinmann, Peter R. Galle, Tim Zimmermann

**Affiliations:** 1st Department of Medicine, University Medical Center Mainz, Langenbeckstr. 1, Mainz, 55131 Germany; University Medical Center Mainz, Institute of Medical Biostatistics, Epidemiology and Informatics, Obere Zahlbacher Str. 69, Mainz, 55131 Germany; Department of General-, Visceral- and Transplantation Surgery, University Medical Center Mainz, Langenbeckstr. 1, Mainz, 55131 Germany; Department of Pathology, University Medical Center Mainz, Langenbeckstr. 1, Mainz, 55131 Germany

**Keywords:** Hepatocellular carcinoma, HCC, OCT1, *SLC22A1*, Biomarker, Sorafenib

## Abstract

**Background:**

The polyspecific organ cation transporter 1 (OCT1) is one of the most important active influx pumps for drugs like the kinase inhibitor sorafenib. The aim of this retrospective study was the definition of the role of intratumoral OCT1 mRNA expression in hepatocellular carcinoma (HCC) as a biomarker in systemic treatment with sorafenib.

**Methods:**

OCT1 mRNA expression levels were determined in biopsies from 60 primary human HCC by real time PCR. The data was retrospectively correlated with clinical parameters.

**Results:**

Intratumoral OCT1 mRNA expression is a significant positive prognostic factor for patients treated with sorafenib according to Cox regression analysis (HR 0.653, 95 %-CI 0.430-0.992; *p* = 0.046). Under treatment with sorafenib, a survival benefit could be shown using the lower quartile of intratumoral OCT1 expression as a cut-off. Macrovascular invasion (MVI) was slightly more frequent in patients with low OCT1 mRNA expression (*p* = 0.037). Treatment-induced AFP response was not associated with intratumoral OCT1 mRNA expression levels (*p* = 0.633).

**Conclusions:**

This study indicates a promising role for intratumoral OCT1 mRNA expression as a prognostic biomarker in therapeutic algorithms in HCC. Further prospective studies are warranted on this topic.

## Background

Hepatocellular carcinoma (HCC) belongs to the most common human cancer entities and shows an increasing incidence [1, 2]. With an estimated 5-year-survival rate of 15 % the prognosis of HCC patients is poor [3]. Curative treatment options are only available for early tumor stages. In particular, patients with a multifocal tumor growth are facing a poor prognosis. Classical chemotherapeutic approaches are largely inefficient due to a pronounced chemoresistance [4]. To date, the oral multikinase inhibitor sorafenib is the standard systemic treatment for patients with advanced HCC [2]. The SHARP trial showed an increase in the median overall survival of about 3 months in the sorafenib treatment group [5]. The effects of sorafenib were slightly weaker in a phase III trial in an asia-pacific population with a more advanced disease [6]. Unfortunately, a substantial fraction of patients faces serious drug-related adverse events under sorafenib treatment that can even result in drug discontinuation. Diarrhea and hand-foot skin reaction are the most common reactions and occur in about 8–16 % [5, 6]. Moreover, there are controversial assumptions regarding the cost effectiveness of sorafenib treatment [7, 8]. These findings underscore the urgent need for biomarkers predicting prognosis and response under treatment with sorafenib. However, convincing biomarkers for the identification of patients that will most likely have a benefit from a systemic treatment with sorafenib are still not defined [9].

The organic cation transporter OCT1 (gene symbol *SLC22A1*) belongs to the amphiphilic solute facilitator (ASF) family of integral transmembrane proteins [10]. It is located at the basolateral membrane of hepatocytes [11]. The physiologic role of OCT1 is the uptake of a broad range of endogenous (e. g. catecholamines and prostaglandins) and exogenous substrates including anticancer drugs like tyrosine kinase inhibitors (e. g. sorafenib) [11–13]. We could show previously that intratumoral downregulation of OCT1 correlates with a worse survival in HCC [10]. In addition, a high pretherapeutic OCT1 expression predicts a complete molecular response to the tyrosine kinase inhibitor imatinib in chronic myeloid leukemia (CML) [14]. It is known that a reduced or aberrant OCT1 expression prevents a sufficient intracellular sorafenib concentration [13].

It was the aim of this retrospective study to define whether OCT1 mRNA expression is a useful biomarker in the systemic therapy of HCC with sorafenib.

## Methods

### Patient characteristics and tissue samples

Clinical data and tumor samples of 60 patients that underwent liver biopsy at the University Medical Center Mainz between January 2001 and December 2013 were analyzed in this study. Clinical and pathological characteristics of this cohort are summarized in Table 1. Primary inclusion criteria were liver biopsy, treatment with sorafenib and registration in the HCC database Mainz. Main exclusion criteria were insufficient RNA-extraction from liver tissue and curative liver transplantation without post-transplant tumor recurrence. All HCC were histologically confirmed. This study was approved by the ethics committee of the local medical board Rhineland-Palatinate and was conducted according to the ethical guidelines of the Declaration of Helsinki. Written informed consent was given by each patient. The liver tissues analyzed in this study were embedded in paraffin. For the evaluation of an AFP response, only patients with AFP levels > 20 ng/ml (AFP-positive HCC) were included. Due to the retrospective approach, AFP response was determined individually at variable time points after initiation of sorafenib treatment.

**Table 1:** Patients and tumor characteristics

Characteristics		
n		60
Gender		
male		54
female		6
Mean age		
years (standard deviation)		64.8 (10.7)
Underlying disease		
alcohol		16
HBV		11
HCV		12
steatosis or NASH		5
others		11
unknown		5
Prior HCC treatment		
yes		35
no		25
Tumor grading		
G1		13
G2		34
G3		8
unknown		5
Tumor burden		
MVI	absent	31
	present	29
EHS	absent	17
	present	43
MVI and/or EHS	absent	6
	present	54
BCLC classification		
A		1
B		1
C		50
D		6
unknown		2
ECOG PS		
0		11
1		39
2		5
3		2
unknown		3
Child-Pugh		
A		14
B		24
C		4
unknown		18
Ascites		
absent		23
present		19
unknown		18
Baseline AFP (ng/ml)		
≤20		22
>20		36
unknown		2

### RNA isolation, RT-PCR and real-time RT-PCR analysis

Paraffin embedded tissue sections of 5-10 μm thickness were used for RNA isolation. Hemo-De solvent (Scientific Safety Solvents, Keller, USA) and the High Pure RNA Paraffin Kit (Roche, Mannheim, Germany) were used for deparaffinization according to the manufacturer’s recommendations. The iScript cDNA Synthesis kit (Biorad, Munich, Germany) was applied for cDNA synthesis from total RNA according to the manufacturer’s recommendations. Quantification of OCT1 (*SLC22A1*) transcripts was performed by real-time PCR. Quantitect SYBR Green PCR Kit (QIAGEN, Hilden, Germany) and validated primers of a Quantitect Primer Assay with the primer sets Hs_SLC22A1_1_SG (QT00019572) and Hs_GAPDH_2_SG (QT01192646) were used according to the manufacturer’s recommendations (QIAGEN, Hilden, Germany). Primer sequences are considered commercially sensitive by the manufacturer and cannot be published. For the amplification, an initial denaturation (15 min at 95 °C) followed by 50 cycles of denaturation (15 s at 94 °C), annealing (30 s at 55 °C), and elongation (30 s at 72 °C). A LightCycler® 480 real-time PCR system (Roche, Mannheim, Germany) was used. Relative expression level of OCT1 (SLC22A1) was calculated by normalization to GAPDH gene expression using LightCycler® 480 software version 1.5.0.

### Statistical analysis

Statistical analyses were performed using SPSS (IBM® SPSS® 21 version 21.0.0.1). For descriptive analyses, mean and standard deviation were calculated for continuous variables. In addition, absolute and relative frequencies were computed for categorical variables. Quantitative, normally distributed variables were analyzed using the unpaired t-test. For the analysis of categorical variables, we used Fisher’s exact test or Mann–Whitney U test. Survival rates between both OCT1 groups were compared by the log-rank test. For graphical visualization Kaplan-Meier curves are presented. The univariable test results have to be considered as explorative. No adjustments for multiple testing have been done here. *P*-values are given for descriptive reasons only. A multivariable Cox regression model adjusted for age was performed for confirmatory analysis with a significance level of 5 %. Hazard ratios with their corresponding *p*-values and 95 % confidence limits are presented.

## Results

### Expression of OCT1 (SLC22A1) mRNA in HCC biopsies

First, we analyzed the intratumoral OCT1 mRNA expression levels. The relative OCT1 expression levels in HCC tissue ranged between 0.0037 and 9.711 with a lower quartile of 0.227.

### Survival according to intratumoral OCT1 mRNA expression

**Fig. 1:**
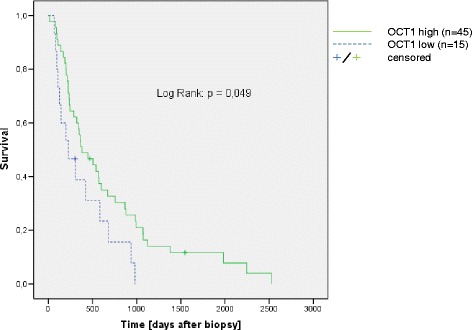
Survival according to the intratumoral OCT1 expression. Patient groups were compared by lower quartile of intratumoral OCT1 expression according to a sensitivity analysis

**Fig. 2:**
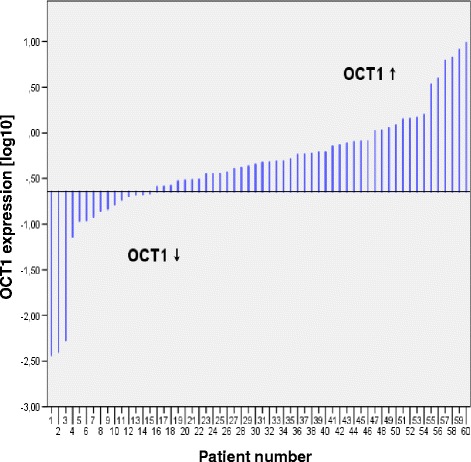
Intratumoral OCT1 expression according to median. The patients were sorted by intratumoral OCT1 expression (*n* = 60). Two patient groups were defined according to the lower quartile of intratumoral OCT1 expression

Cox regression analysis revealed a significant positive association between OCT1 mRNA expression level and patient survival in patients treated with sorafenib (HR 0.653; 95 %-CI 0.430-0.992; *p* = 0.046; Table 2). Patient age at beginning of sorafenib treatment did not have a significant impact (*p* = 0.144). As the majority of patients in this cohort were male, the variable gender was excluded in the cox regression analysis. A sensitivity analysis showed a slight but relevant survival benefit in the univariable log-rank test using the lower quartile of OCT1 mRNA expression as a cutoff (*p* = 0.049; Fig. 1). According to the sensitivity analysis, patients were subdivided into two groups by the intratumoral OCT1 mRNA expression level (<lower quartile vs. ≥ lower quartile, Fig. 2).

**Table 2:** Cox regression

	HR	95 %-CI	*P*-value
OCT1 expression level [log10]	0.653	0.430 – 0.992	0.046
age (begin sorafenib) [years]	0.980	0.955 – 1.007	0.144

### OCT1 mRNA expression in correlation with patient and tumor characteristics

Patients and tumor characteristics are listed in Table 1. No differences between the two groups (OCT1 mRNA expression < lower quartile vs. ≥lower quartile) could be shown regarding formerly described relevant baseline characteristics like presence of ascites (*p* = 0.504), Barcelona-Liver Cancer Clinic stage (BCLC stage, *p* = 0.988), and Eastern Cooperative Oncology Group Performance status (ECOG; *p* = 0.099, Table 3). Macrovascular invasion (MVI) was slightly more frequent in the group showing a low OCT1 mRNA expression (*p* = 0.037, Table 3). Prior HCC treatment did not have a statistically significant impact.

**Table 3:** Patients and tumor characteristics according to the intratumoral OCT1 mRNA expression

Characteristics	OCT1 (*SLC22A1*)	OCT1 (*SLC22A1*)	*P*-value
	Low expression	High expression	
	(< lower quartile)	(≥ lower quartile)	
*n*	15	45	
Gender			
male	14	40	
female	1	5	1.000 (Fisher’s exact test)
Mean age			
years (standard deviation)	65.022 (7.190)	64.658 (11.667)	0.910 (unpaired t test)
Underlying liver disease			
alcohol	2	14	
HBV	3	8	
HCV	4	8	
steatosis or NASH	0	5	
others	5	6	
unknown	1	4	0.224 (Fisher’s exact test)
Prior HCC treatment			
yes	6	29	
no	9	16	0.133 (Fisher’s exact test)
Tumor grading			
G1	3	10	
G2	8	26	
G3	4	4	
unknown	0	5	0.265 (Mann–Whitney U test)
Tumor burden			
MVI absent	4	27	
present	11	18	0.037 (Fisher’s exact test)
EHS absent	5	12	
present	10	33	0.743 (Fisher’s exact test)
MVI			
and/or EHS absent	1	5	
present	14	40	1.000 (Fisher’s exact test)
BCLC classification			
A	0	1	
B	1	0	
C	12	38	
D	2	4	
unknown	0	2	0.988 (Mann–Whitney U test)
ECOG PS			
0	2	9	
1	8	31	
2	2	3	
3	2	0	
unknown	1	2	0.099 (Mann–Whitney U test)
Child-Pugh			
A	1	13	
B	11	13	
C	0	4	
unkown	3	15	0.195 (Mann–Whitney U test)
Ascites			
absent	5	18	
present	6	13	
unknown	4	14	0.504 (Fisher’s exact test)
Baseline AFP (ng/ml)			
≤20	2	20	
>20	11	25	
unknown	2	0	0.103 (Fisher’s exact test)
mean duration			
sorafenib treatment			
days (standard deviation)	161 (126)	149 (128)	0.764 (unpaired t test)

### AFP response according to the intratumoral OCT1 mRNA expression

For the evaluation of the AFP response, only patients with AFP levels >20 ng/ml (AFP-positive HCC) were included in the analysis. Patients were only categorized as AFP responders if a reduction in AFP levels of at least 20 % was achieved under treatment with sorafenib [15, 16]. Table 4 shows the AFP response of the AFP positive patients in this cohort according to the OCT1 mRNA expression (<lower quartile vs. ≥lower quartile; *n* = 36). Concerning the AFP response under treatment with sorafenib, there were no differences between the OCT1 mRNA low and the OCT1 mRNA high expression groups in this cohort (*p* = 0.633, Table 4).

**Table 4:** AFP response

Baseline AFP (ng/ml) >20	OCT1 (*SLC22A1*)	OCT1 (*SLC22A1*)	*P*-value
	Low expression	High expression	
	(< lower quartile)	(≥ lower quartile)	
n	11	25	
AFP responders	1	6	
AFP non-responders	7	14	
unknown	3	5	0.633 (Fisher’s exact test)

## Discussion

Intratumoral downregulation of OCT1 in HCC has been described by us and others [10, 13]. In a previous work we showed that down-regulation of OCT1 is associated with reduced survival in patients that underwent liver resection or transplantation [10]. Whether reduced intratumoral OCT1 mRNA expression assessed from tumor biopsies is of prognostic value under sorafenib treatment has not been defined yet. We performed this retrospective study as the identification of novel biomarkers in HCC treatment is of special interest in terms of individualized medicine.

For this analysis, OCT1 mRNA was quantified with a commercially available primer set that has been comprehensively validated and correlated with OCT1 protein expression by our group [10, 17]. OCT1 exhibits SNPs that might affect OCT1 function. In the background of CML, several studies investigated the association between OCT1 SNPs and clinical outcome with contradictory results [18–21]. Importantly, one study suggests that contradictory results might be due to interference between SNPs and primer sites [19]. Upon request, the manufacturer of the primer assays used in this study ensured that the primer sites do not interfere with the most relevant SNPs as proposed by Giannoudis et al. [19]. A sensitivity analysis revealed that particularly patients with a baseline OCT1 mRNA expression within the range of the lower quartile have a significantly impaired survival under treatment with sorafenib. The poor prognosis under treatment might be at least in part explained by a reduced OCT1-mediated drug uptake due to non-functional, truncated proteins [13].

This retrospective analysis shows that a reduced intratumoral OCT1 mRNA expression results in a worse survival in patients treated with sorafenib. This effect is independent of other strong prognostic factors like the presence of ascites, BCLC stage and ECOG performance status [22]. A correlation between the prognostically unfavorable low intratumoral OCT1 expression and MVI could be shown here if the lower quartile of OCT1 expression was used as a cutoff (*p* = 0.037). This correlation is not significant if the cutoff is median OCT1 expression (*p* = 0.120, data not shown). Also in previous studies using median OCT1 expression as a cutoff, a statistically significant correlation between OCT1 expression and MVI was not shown [10]. The impact of this observation will be further analyzed in a subsequent study.

The prognostic role of tumor markers like AFP in HCC has been studied extensively [23]. Previous studies showed that AFP response was significantly associated with the overall survival also in patients with advanced HCC treated with sorafenib [16]. Probably due to variable times of AFP measurement in this retrospective analysis we could not reproduce this finding in the context of OCT1 mRNA expression levels.

A limitation of the current study is the retrospective nature of data collection. The biopsies were acquired in context of primary diagnosis of the HCC. Variations in tumor genetics may occur during the course of disease [24]. In addition, the time frame between biopsy acquisition and beginning of sorafenib treatment varies. Due to this fact, a lead time bias and effects on the basis of variable stages of tumor spread should be considered [25]. Some patients enrolled in this analysis have been treated in the early phase after approval of sorafenib. Initially, few patients with reduced liver function and performance status were treated with sorafenib. To date, guidelines do not recommend the use of sorafenib in these patients [2]. As common for retrospective trials, the reliability and validity of patient’s report in terms of adherence to medication remains unknown [26]. Radiological response could not be correlated with OCT1 mRNA expression levels in this cohort due to a lack of data.

Although the acquisition of HCC tissue via transcutaneous biopsy is a feasible method with a good risk-benefit ratio, it should be considered that intratumoral heterogeneity in OCT1 mRNA expression might occur. The alternative approach of a HCC resection remains reserved to a relatively small fraction of patients [2]. However, facing all the drawbacks, the identification of patient subgroups with the best response to an antitumor agent in HCC by information drawn from tumor biopsies is still a promising approach.

## Conclusions

The identification of novel biomarkers for anticancer therapy is of particular importance in terms of prevention of side effects caused by therapeutics with limited efficacy in the individual patient as well as for economic reasons. This study shows that intratumoral OCT1 mRNA expression might play a role as a prognostic biomarker in sorafenib-based HCC therapy. Further, prospective trails are warranted on this topic.

## Abbreviations

MVI: macrovascular invasion
EHS: extrahepatic spread
AFP: alpha-fetoprotein
